# A Novel Case of Concomitant PHEX and ALPL Mutation In a Family With Rickets

**DOI:** 10.1210/jcemcr/luad151

**Published:** 2023-12-07

**Authors:** Carmen Polanco Santos, Juana Cordero Garate, Leila Zeinab Khan

**Affiliations:** Endocrinology and Metabolism Institute, Cleveland Clinic Foundation, Cleveland, OH 44195, USA; Jacobi Medical Center, Albert Einstein College of Medicine, Bronx, NY 10461, USA; Endocrinology and Metabolism Institute, Cleveland Clinic Foundation, Cleveland, OH 44195, USA

**Keywords:** rickets, hypophosphatemia, hypophosphatasia, *PHEX*, *ALPL*, burosumab-twsa

## Abstract

Currently, no published cases report concomitant X-linked hypophosphatemia (XLH) and adult hypophosphatasia (HPP). Both diseases share clinical phenotypes that are almost indistinguishable. The correct diagnosis may be missed without a standardized laboratory and genetic testing approach. Pathogenic variants in the phosphate regulating endopeptidases homolog X-linked gene (*PHEX*) and the tissue-nonspecific alkaline phosphatase gene (*ALPL*) are genes that cause XLH and HPP, respectively. We describe a concomitant yet undescribed genetic pathogenic variant in a family. A 61-year-old woman was referred by orthopedic surgery for the presence of bilateral leg bowing and short stature during the assessment of knee surgery. The patient had a biochemical workup relevant for low serum phosphorus and 1,25-dihydroxy vitamin D and normal alkaline phosphatase (ALP). Genetic analysis revealed pathogenic variants in *PHEX* and *ALPL*. Her 42-year-old daughter shared identical symptoms and genetic variants with her mother. Both patients started conventional treatment for XLH with phosphorus and vitamin D, and the daughter later switched to burosumab-twza. Adult XLH and HPP may have similarities in clinical presentation but differ in some essential laboratory findings. Normal ALP levels helped direct our diagnosis toward XLH. However, the diagnosis was challenging due to the presence of concurrent variants in the genes involved. These variants illustrate the significant heterogeneity of the clinical expression.

## Introduction

Inherited bone mineralization disorders comprise a heterogeneous group of diseases that differ vastly in origin but present similarly with rickets and dental problems. These clinical signs are common features shared. Laboratory and genetic tests help guide the analysis; however, the diagnosis may be more challenging when concomitant pathogenic variants exist.

X-linked hypophosphatemia (XLH) is an inherited disease of phosphate metabolism with an incidence estimated at 3.9 per 100 000 live births (1). It is caused by a loss-of-function pathogenic variants in the *PHEX* gene, which is predominantly expressed in osteoblasts and reduces serum levels of fibroblast growth factor 23 (FGF23) (2). Elevated levels of serum FGF23 affect phosphate levels in 2 main ways: (1) increasing urinary phosphate excretion and (2) limiting intestinal phosphate absorption. The first is by downregulating the sodium-phosphate transporters in the distal renal tubule and the latter by restricting calcitriol synthesis to abnormally low or normal levels despite hypophosphatemia (2, 3). XLH frequently manifests in the early years of life; however, diagnosis can also be delayed until adulthood as in this case. In adults, joint pain, dental abscesses, and impaired mobility may be the initial presenting complaints (4).

Hypophosphatasia (HPP) is a rare inborn error caused due to loss of function pathogenic variants within the gene that encodes the tissue-nonspecific isoenzyme of serum alkaline phosphatase. It is characterized by low serum alkaline phosphatase (ALP) activity (hypophosphatasaemia) and is classified into different groups according to the age of onset and severity (5). The overall incidence of the various forms of HPP is poorly understood, and prevalence is estimated at 1/100 000 (6). Patients with adult-onset HPP experience musculoskeletal pain, early loss of teeth, pseudogout, and osteomalacia (7).

We present a familial case of a 61-year-old woman and her 42-year-old daughter with rickets in the presence of rare concomitant *PHEX* and *ALPL* genetic pathogenic variants that have never been previously described.

## Case Presentation

A 61-year-old Vietnamese woman was referred to the endocrinology clinic by orthopedic surgery for osteomalacia evaluation before knee surgery for severe osteoarthritis. The patient reported a long history of progressive joint pain, especially in the knees, making walking difficult. She presented with short stature in addition to bowing of the legs and genu valgum, which she mentioned had been present since childhood; an assessment for rickets was never done. She had been treated with frequent steroid injections in both of her knees. The patient took gabapentin; acetaminophen; calcium 600 mg with vitamin D 400 units, 2 daily tablets; and naproxen for joint pain with minimal relief. Her past medical history revealed several dental abscesses and permanent teeth loss by age 40 but no record of fractures or kidney stones. She had a family history of osteoporosis in her mother and rickets in her sister. The physical examination was notable for weight of 45.7 kg, height of 138.4 cm, a body mass index of 24 kg/m^2^, head with frontal bossing, oral cavity with complete dentures, normal upper extremity, bowed legs left > right and genu valgum but with no alterations of the feet.

## Diagnostic Assessment

Initial laboratory analysis revealed: phosphate 2 mg/dL (0.64 mmol/L) (reference range: 2.7-4.8 mg/dL; 0.87-1.5 mmol/L), calcium 9.3 mg/dL (2.3 mmol/L) (reference range: 8-10.2 mg/dL; 2-2.6 mmol/L), PTH 32.3 pg/mL (3.4 pmol/L) (reference range: 19-88 pg/mL; 2-9.3 pmol/L), ALP 128 U/L (2.1 mckat/L) (reference range: 24-123 U/L; 0.4-2.1 mckat/L), 25-hydroxyvitamin D 50 ng/mL (125 nmol/L) (reference range: 31-80 ng/mL; 77-199 nmol/L), 1,25-hydroxyvitamin D 8.4 pg/mL (21.8 pmol/L) (reference range: 15-60; 39-156 pmol/L) urine phosphate 660 mg/d (21 mmol/d) (normal range: 400-1300 mg/d, 12.9 to 42.0 mmol/day), urine calcium 86 mg/d (2.1 mmol/d) (reference range: 100-300, 2.5-7.5 mmol/d). The ratio of maximum tubular reabsorption of phosphate to glomerular filtration rate was low, with a value of 1.96 mg/dL (normal range 2.2-3.4 mg/dL). X-rays revealed severe knee osteoarthritis and diffuse demineralization. A bone density scan showed osteopenia at the wrist.

Based on these results, the patient started treatment with calcium carbonate 600 mg plus vitamin D3 400 units, 1 tablet orally 3 times daily, and phosphorus 250 mg tablet once daily. On her subsequent follow-up, after 3 months of initial treatment, phosphorus levels remained low (Table 1) with no improvement in joint, bone pain, or energy level. Genetic counseling and genetic sequencing were performed and revealed 2 likely pathogenic variants: c.304G>T (p.Gly102Trp), heterozygous in *PHEX*, and c.1402G>T (p.Ala468Ser) heterozygous in *ALPL*; both were novel pathogenic variants. The mutations were found in the Invitae Diagnostic Database. Additional confirmation would be helpful with a serum pyridoxal 5′-phosphate concentration especially since the alkaline phosphatase levels were not decreased as one would typically suspect.

**Table 1.** Mother blood tests before and after conventional treatment

	August 2021 Before initial treatment	January 2022 3m after treatment	April 2022 6m after treatment	Normal range
Inorganic phosphate	2.0 mg/dl (0.64 mmol/L)	2.9 mg/dl (0.92 mmol/L)	2.4 mg/dl (0.76 mmol/L)	2.7-4.8 mg/dl (0.87-1.5 mmol/L)
Calcium	9.3 mg/dl (2.3 mmol/L)	9.5 mg/dl (2.37 mmol/L)	9.7 mg/dl (2.42 mmol/L)	8.5-10.2 mg/dl (2 -2.6 mmol/L)
PTH	32.3 pg/ml (3.4 pmol/L)	53 pg/ml (5.3 pmol/L)	40 pg/ml (4.0 pmol/L)	19 – 88 pg/ml (2-9.3 pmol/L)
Alkaline phosphatase	128 iu/l (2.1 mckat/L)	131 iu/l (2.2 mckat/L)	130 iu/l (2.2 mckat/L)	34-123 iu/l (0.4-2.1 mckat/L)
25-hydroxyvitamin D	50.0 ng/ml (125 nmol/L)			31-80 ng/ml (77-199 nmol/L)
1 25-dihydroxy Vitamin D	8.4 pg/ml (21.8 pmol/L)	25.8 pg/ml ( 61.9 pmol/L)		15-60 pg/ml (39-156 pmol/L)
Calcium urine	86.9 mg/d (2.1 mmol/d)	139 mg/d (3.4 mmol/d)	112 mg/d (2.8 mmol/d)	100 – 300 mg/d (2.5-7.5 mmol/d)

Values in parenthesis are International System of Units (SI)

After the genetic results, her 35-year-old daughter presented to the endocrinology clinic for evaluation. She reported longstanding bowing of the legs, knee osteoarthritis, fatigue, and muscle pain. She had regular teeth development and denied any history of fractures. Her physical examination showed short stature with a height of 135.3 cm and tenderness in extremities with bowing of the legs and genu valgus. Radiologic findings showed mild lateral bowing of the tibia and fibula, degenerative changes at the knee joint, and mild bilateral hallux valgus. Laboratory analyses revealed phosphate 2.1 mg/dL (0.67 mmol/L) (reference range: 2.7-4.8 mg/dL; 0.87-1.5 mmol/L), calcium 9.3 mg/dL (2.3 mmol/L) (reference range: 8-10.2 mg/dL; 2-2.6 mmol/L), PTH 47 pg/mL (4.9 pmol/L) (reference range: 19-88 pg/mL; 2-9.3 pmol/L), ALP 89 U/L (1.4 mckat/L) (reference range: 24-123 U/L; 0.4-2.1 mckat/L), 25-hydroxyvitamin D 47 ng/mL (117 nmol/L) (reference range: 31-80 ng/mL; 77-199 nmol/L), 1,25-hydroxyvitamin D 28 pg/mL (67.2 pmol/L) (reference range: 15-60; 39-156 pmol/L), urine phosphate 668 mg/d (20.7 mmol/d) (reference range: 400-1300 mg/d, 12.9 to 42.0 mmol/day), urine calcium 95 mg/24 hour (reference range: 100-300, 2.5-7.5 mmol/d). The ratio of maximum tubular reabsorption of phosphate to glomerular filtration rate was low, with a value of 2.09 mg/dL (normal range 2.2-3.4 mg/dL). Genetic analysis revealed the same 2 pathogenic variants as her mother. Interestingly, during the workup of rickets, the patient had acute cholecystitis requiring surgery, and the ALP levels never increased above the upper reference range, as typically observed in an episode of cholecystitis.

## Treatment

Given the *PHEX* genetic variant and low serum phosphorus level in combination with normal ALP (absence of low serum ALP, characteristic of HPP), the patient started treatment for XLH. The mother and daughter received conventional treatment—phosphorus (combination of potassium phosphate and sodium phosphate) plus calcitriol—with oral phosphorus to 250 mg, 1 tablet daily, and calcitriol 0.25 mcg twice daily for the first and oral phosphorus to 250 mg and calcitriol 0.25 mcg capsule twice daily for the latter.

## Outcome and Follow-up

**Figure 1.**
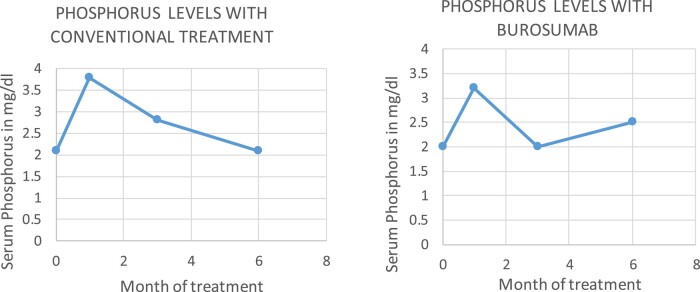
Daughter serum phosphorus levels.

At the subsequent follow-up, low phosphorus levels persisted, but there was a slight improvement in bone and joint pain in both mother and daughter. The dose of oral phosphorus was increased to 250 mg 3 times per day, and we started the submission approval for burosumab-twza to avoid the adverse effects of high phosphate treatment. Burosumab-twza was approved for the daughter. She began with injections of 1 mg/Kg (60 mg) every 4 weeks (conventional treatment was withdrawn 1 week before). After 2 weeks of treatment, her fasting phosphorus level was 3.8 mg/dL (1.22 mmol/L), and after 3 months (after her third injection), the fasting lab was 2.8 mg/dL (0.90 mmol/L) (Fig. 1). On her last follow-up, 6 months after burosumab-twza initiation, her serum phosphorus level was 2 mg/dL, and she reported a significant decrease in pain and stiffness and a significant improvement in physical function and energy.

The mother decided to continue conventional treatment due to frequent travel lasting more than 6 months. While on conventional therapy with phosphorus neutral tablets (a combination of potassium and sodium phosphate) and calcitriol, she reported improvement in joint pain and fatigue, but unfortunately her follow-up has been complicated by her long time out of the country. The last serum phosphorus level was 2.4 mg/dL. We plan to discuss the transition to burosumab-twza in the future.

## Discussion

In the present cases, the patients were diagnosed with XLH. The absence of persistent hypophosphatasemia, the hallmark for diagnosing all forms of HPP, ruled out a pathogenic variant in *ALPL*. There is considerable overlap between signs and symptoms of adults with XLH and HPP; rickets, osteomalacia, odontodysplasia, enthesopathy, and arthropathy are common in the clinical presentation. Differentiation can be made with the help of laboratory tests. In Table 2, we can see the main laboratory differences between adult XLH and HPP. It is essential to mention that laboratory values may vary, and the diagnosis of each disease should be based on a combination of clinical tests, biochemical findings, family history, and, whenever possible, genetic studies.

**Table 2.** Laboratory differences between XLH and HPP

XLH	HPP
Low phosphorus	High phosphorus
High-normal ALP	Low ALP
Normal PTH	Low PTH
Normal calcium	Hypercalcemia
Hyperphosphaturia	Hypercalciuria

Abbreviations: ALP, alkaline phosphatase; HPP, hypophosphatasia; XLH, X-linked hypophosphatemia.

The role of treatment has yet to be well studied, and therapy is reserved for individuals with symptoms, biochemical evidence of osteomalacia, recurrent pseudofractures, or stress fractures (8). We started treatment with phosphorus and calcitriol for 6 months but switched to burosumab-twza due to a lack of improvement in the daughter's symptoms and to avoid the adverse effects of conventional treatment such as nephrocalcinosis and hyperparathyroidism (8). Burosumab-twza is a human anti-FGF23 monoclonal antibody that directly inhibits the activity of FGF23. Thereby correcting the aberrant phosphate homeostasis (9). Randomized controlled trial results demonstrated that with continued exposure to burosumab-twza, phosphorus homeostasis is maintained and the proportion of fully healed fractures and pseudofractures progressively increase. The pain, stiffness, and functional exercise capacity scores improve tremendously. It is the only approved treatment for XLH (10).

Although there are many similarities in patients with bone mineralization disorders, efforts to find the differences between these diseases must be comprehensive to provide appropriate treatment. A careful clinical history, laboratory analysis, and genetic correlation are essential for a correct diagnosis. Early treatment is a crucial factor in slowing the progression of the disease. As in the case of our patients, historically, many patients with XLH or HPP have not received medical care as they progressed through adolescence and into adulthood. Transitioning care from pediatric to adult providers is essential for patients with XLH, and transition programs should be developed.

## Learning Points

- For patients with bone mineralization disorders, a careful clinical history correlating biochemical, phenotypic, and genetic findings is essential for a correct diagnosis.
- The hallmark for the diagnosis of all forms of HPP is persistent hypophosphatasemia.
- An interdisciplinary approach is paramount in patients with XLH and HPP, with special mention paid to dental, orthopedic, and psychological care.
- Early treatment with burosumab-twza in XLH is a crucial factor in slowing the progression of the disease.
- As caregivers, we can guide patients and encourage them to be actively involved in their care to decrease the lifelong impact that XLH and HPP can have.

## Contributors

All authors made individual contributions to authorship C.P.S. was involved in the diagnosis and management of this patient and C.P.S, J.C.G, and L.Z.K in manuscript submission. All authors reviewed and approved the final draft.

## Data Availability

Original data generated and analyzed during this study are included in this published article.
